# Supplementary material for: Asynchronous magnetic resonance elastography: Shear wave speed reconstruction using noise correlation of incoherent waves
Source: Magn Reson Med. 2022 Oct 27;89(3):990–1001. doi: 10.1002/mrm.29502 (PMC9792433; doi:10.1002/mrm.29502)
Supplement: Supplementary file 1 — DATA S1 MATLAB code used to generate the numerical simulations of Figure 1 [file MRM-89-990-s002.zip › k-Wave/helpfiles/example_tvsp_equivalent_source_holography.html]

Equivalent Source Holography Example (k-Wave)


# Equivalent Source Holography Example

This examples demonstrates how to project a plane of measured data using `kspaceFirstOrder3D` by calculating an equivalent mass source (i.e., `source.p` with `source.p_mode =
’additive’`) positioned in a parallel plane that recreates the measured data. It builds on the Simulations In Three Dimensions and Holographic Projections Using The Angular Spectrum Method examples.

- Open the file in the MATLAB Editor
- Run the file in MATLAB

For a more detailed discussion of this example and the underlying techniques, see B. Treeby, F. Lucka, E. Martin, E. and B. Cox, "Equivalent-Source Acoustic Holography for Projecting Measured Ultrasound Fields Through Complex Media," *IEEE Transactions on Ultrasonics, Ferroelectrics, and Frequency Control*, vol. 65, no, 10, pp.1857-1864, 2018.

## Contents

- Overview
- Generating the input and output data
- Calculating the equivalent source
- Holographic projections

## Overview

Holographic projections of experimental ultrasound measurements generally use the angular spectrum method or Rayleigh integral, where the measured acoustic pressure is imposed as a Dirichlet boundary condition. In contrast, the acoustic modelling functions in k-Wave use interior mass sources (i.e., `source.p`) to introduce acoustic energy into the simulation. It is possible to enforce measured values in the simulation by using the `source.p_mode =
’dirichlet’` option. At each time step, this replaces the pressure values over the measurement plane with the measured data. However, this data replacement at discrete time steps does not exactly preserve the spatial gradients that existed in the original field and thus introduces numerical errors. (Note, this is not unique to k-Wave and also applies to other collocation methods including those based on finite differences.)

In this example, the function `calculateMassSourceCW` is used to generate an equivalent interior mass source, positioned on a parallel plane, that reproduces the measurement data. The function uses gradient-based optimization to find the equivalent source, where the update steps are calculated using gradient descent and the gradients of the error functional are calculated using a numerical forward model and its adjoint (more details are given in the reference above). The calculated equivalent-source can then be used with `kspaceFirstOrder3D` to accurately compute holographic projections through complex media including nonlinearity and heterogeneous material properties. Note, for fields that are not time-harmonic, the function `calculateMassSource` should be used.


## Generating the input and output data

The function `kspaceFirstOrder3D` is used to simulate the output from a rectangular piston transducer driven by a 2 MHz continuous wave sinusoid created using `createCWSignals`. The transducer is oriented in the z-direction. The sensor mask is defined as two planes: an x-y plane positioned 8 grid points from the source (this is used for the input data), and the central x-z plane through the domain (this is used for comparison with the projected fields).

```
% define rectangular source mask
source.p_mask = zeros(Nx, Ny, Nz);
source.p_mask(Nx/2 - rect_x_radius + 1:Nx/2 + rect_x_radius, Ny/2 - rect_y_radius + 1:Ny/2 + rect_y_radius, 1) = 1;

% define source signal as a continuous wave sinusoid
source.p = createCWSignals(kgrid.t_array, source_freq, 1, 0);

% define two planar sensor masks using opposing corners of a cuboid
sensor.mask = [1, 1,    input_plane_z_index, Nx, Ny,   input_plane_z_index; ...
               1, Ny/2, input_plane_z_index, Nx, Ny/2, Nz].';
```

The time step for the simulation is defined using an integer number of points-per-period. The simulation is then run until steady state, with the time-varying acoustic pressure recorded over the last three cycles. After the simulation is complete, the function `extractAmpPhase` is used to extract the amplitude and phase at each point over the two sensor planes. Using an integer number of points-per-period and recording for an integer number of periods means the amplitude and phase can be precisely extracted from the amplitude spectrum without spectral leakage.

```
% compute sampling rates, forcing points-per-period to be an integer
points_per_wavelength = c0 / (source_freq * dx);
points_per_period = round(points_per_wavelength / cfl);

% compute corresponding time spacing
dt = 1 / (points_per_period * source_freq);    

% create the time array
Nt = round(t_end / dt);
kgrid.setTime(Nt, dt);

% set the start time to only record the last three periods
sensor.record_start_index = kgrid.Nt - 3 * points_per_period + 1;
```

The calculated input plane is shown below (referred to herein as the measured data). Note, to project experimental data, this can be replaced with a 2D matrix of complex pressure values extracted from the experimentally measured data.


## Calculating the equivalent source

The function `calculateMassSourceCW` is used to calculate the equivalent source. This computes a continuous wave (CW) additive pressure source positioned on a plane parallel to the measurement plane separated by `source_offset` grid points (`calculateMassSource` should be should be used for non-CW data). For focused ultrasound fields, the equivalent source plane will usually need to be larger than the measured input plane. In this case, the `grid_expansion` input should be specified so that the grid size is large enough to capture the source variation. Note, the position of the equivalent source plane does not need to match the position of the real source in the experiment (although this can be useful in some cases). It is merely a device to generate the correct input to the numerical model.

```
% calculate equivalent source
[source_estimate, optim_params] = calculateMassSourceCW(input_plane_complex, dx, source_freq, c0, source_offset, grid_expansion);
```

As `calculateMassSourceCW` runs, a graphical output is provided showing the progress of the optimisation. This shows the amplitude and phase of the input (measured) data, the corresponding amplitude and phase calculated using the current estimate of the equivalent source, the current amplitude and phase error, and a convergence plot of the error with iteration number. By default, 20 iterations of the optimisation are performed (where two model runs are needed per iteration). In most cases, this will be sufficient for the solution to have converged. If needed, the number of iterations can be controlled using the optional input parameter `'NumSteps'`. For this example, after 20 iterations, the error is very small, and there is a close match between the measured and modelled data.

After the specified number of iterations, the last estimate of the equivalent source is returned. This is shown below. Note, in this example, the amplitude of the equivalent source does not conform to an exact rectangle. This is because the input plane where the measured data is recorded is positioned sufficiently far from the source that evanescent waves are not recorded.


## Holographic projections

The acoustic pressure field generated using the original rectangular source is shown below (reference field). For comparison, three relative error plots are also given corresponding to different holographic projections of the input data.

1. The measured input plane projected with `kspaceFirstOrder3D` using `source.p_mode = ’dirichlet’`.
2. The calculated equivalent source plane projected with `kspaceFirstOrder3D` using `source.p_mode = ’additive’` (the default).
3. The measured input plane projected with `angularSpectrumCW`
.

As mentioned above, when directly enforcing the measured data within `kspaceFirstOrder3D` using `source.p_mode = ’dirichlet’`, the spatial gradients that existed in the original field are not exactly preserved, so errors of a few percent are seen in the projected field (Dirichlet projection). These errors are reduced by using the equivalent source and are comparable to using the angular spectrum method (in this case slightly smaller). The calculated equivalent source can also be used to project the measured data through heterogeneous media including the effects of nonlinearity.
